# Research on Factors Affecting Chinese College Students' Marriage Intention: Applying the Theory of Planned Behaviour

**DOI:** 10.3389/fpsyg.2022.868275

**Published:** 2022-06-01

**Authors:** Jianwei Xie, Xiaochang Hong

**Affiliations:** Communist Youth League Committee, Wenzhou Medical University, Wenzhou, China

**Keywords:** marriage intention, attitude towards the behaviour, subjective norm, perceived behavioural control, Chinese college students

## Abstract

With the second demographic transition, the marriage rate in China has decreased annually. This reduction will have a key impact on national economic and social development. There is a need to investigate the factors that influence college students' marriage intentions, which can predict the development trend of marriage and family formation in China's future society to some extent. Previous studies focus on the extraction of college students' beliefs about marriage and love, whereas few studies have investigated how these beliefs affect marriage intention and marriage behaviour. Based on the theory of planned behaviour, this study aims to explore the impact of attitude towards behaviour, subjective norm, and perceived behavioural control on marriage intention; analysed the key predictors of college students' marriage intentions; and provided implications for pro-marriage policy. Using convenience sampling, data were obtained from 772 college students (mean age = 20.38 ± 2.38 years; 64.5% women) from three universities in China. Two types of questionnaires were designed to elicit salient beliefs and marriage intentions, respectively. Students' marriage intentions were positively correlated with attitude towards behaviour, subjective norm, and perceived behavioural control. Most participants were inclined to get married, and the marriage intention of women was significantly lower than that of men. The important beliefs and factors influence students' marriage intention included stereotypical gender identity, low fertility intention, weakened inter-generational transmission of traditional family ideas, materialisation of marriage, and negative information about marriage disseminated by the mass media. To promote college students' marriage intention, pro-marriage policies conducive to fertility, good economic prospects, more employment opportunities, positive publicity of marriage by the mass media are suggested to make, and it is necessary for universities to offer marriage and love courses to college students.

## Introduction

According to statistics from the Civil Affairs Bureau of China, the number of marriage registrations in 2020 was 8.131 million. Compared with the same period of the previous year, the number decreased by 12.2%, reaching its lowest point since 2003. Concurrently, since the 1990s, the phenomenon of late marriage has been prominent in China. People aged 25–29 years have replaced people aged 20–24 years as the main group to get married, and the proportion of marriage registration in the high age group (>40 years) has increased significantly (Dai and Lv, 2021). This reveals that China is experiencing a second demographic transition.

According to the theory of the second demographic transition, in the post-industrial era, with the rise of individualism and the improvement of women's education level, people pursue higher self-worth. Marriage is no longer a necessity, and the driving force to enter marriage is weaker, resulting in a decrease in marriage and birth rates and an increase in divorce, cohabitation, and remarriage (Lesthaeghe, 1995; Van de Kaa, 2001; Raymo et al., 2021). The impact of the second demographic transition on families also includes the separation of marriage and childbirth. Marriage is no longer the premise of childbirth, which also leads to the increase of out of wedlock births in many societies, including the United States and Nordic Countries (Ellwood and Jencks, 2004; Yu and Xie, 2019). However, this phenomenon is not common in China. In addition to the policy restrictions on illegitimate childbearing, the Chinese view of childbearing is more traditional. A complete family is considered a necessary condition for raising children, and marriage and childbearing are still closely linked (Yu and Xie, 2019). Therefore, late and less marriage in China often directly leads to social problems, such as a decline in fertility and an ageing population. At present, the phenomenon of late and less marriage among young people has attracted extensive attention.

In China, the young generation is facing the impact and influence of social transformation. Some young people's expectations for marriage include the superposition of lookism, materialism, romanticism, comparison, and departmentalism (Mu, 2021). The serious imbalance between the number of men and women, as well as high real estate rates and child-rearing costs are also reliable explanations for the current low marriage rate. Young people's marriage behaviour affects social changes (Raymo, 2003; Yabiku, 2004; Nobles and Buttenheim, 2008). If the young generation's marriage intention is kept low, the phenomenon of low fertility and an ageing population in China will become increasingly serious. College students are the backbone of youth groups and an important reserve of national talent. Their view of marriage can predict the development trend of marriage and family formation in China's future society to some extent, meanwhile also affecting China's social stability and economic development. Therefore, helping college students form an appropriate view of marriage and love and facilitating their smooth entry into marriage is a topic of interest for many scholars. Previous research has focused on college students' love attitudes and motivations, mate selection methods and criteria, and education on love and marriage (Zhang, 2019; Zhao, 2019; Kong and Zang, 2021). Most studies focus on the extraction of college students' beliefs about marriage and love, whereas few studies have investigated how these beliefs affect marriage intention and marriage behaviour. The theory of planned behaviour, developed by Ajzen (1991), proposes that individual behaviour intention is an important factor determining whether their actual behaviour occurs; further, this theory has also been supported by several empirical studies. Therefore, based on the theory of planned behaviour, this study explored the influential predictors and mechanisms of college students' marriage intentions and provides implications for pro-marriage policy.

## Theory and Hypotheses

The theory of planned behaviour explains the general decision-making process of individual behaviour from the perspective of information processing and is based on the expected value theory. It claims that individual behaviour is the result of deliberate planning. This theory contributes to understanding how individuals adhere to and change their behavioural patterns (Ajzen, 1991; Duan and Jiang, 2008). Scholars have applied the theory of planned behaviour to different behaviours (Canniere et al., 2009; Ajzen and Klobas, 2013; Jekauc et al., 2015; Normaliza et al., 2018). Most studies have confirmed that this theory can significantly improve the explanatory and predictive power of behaviour. Behaviour intention is considered an antecedent of individual behaviour. It refers to the individual's judgement on the subjective probability of implementing a certain behaviour, which reflects their willingness to implement that behaviour. The stronger the behavioural intention, the greater the probability of implementing a certain behaviour.

**Figure 1:**
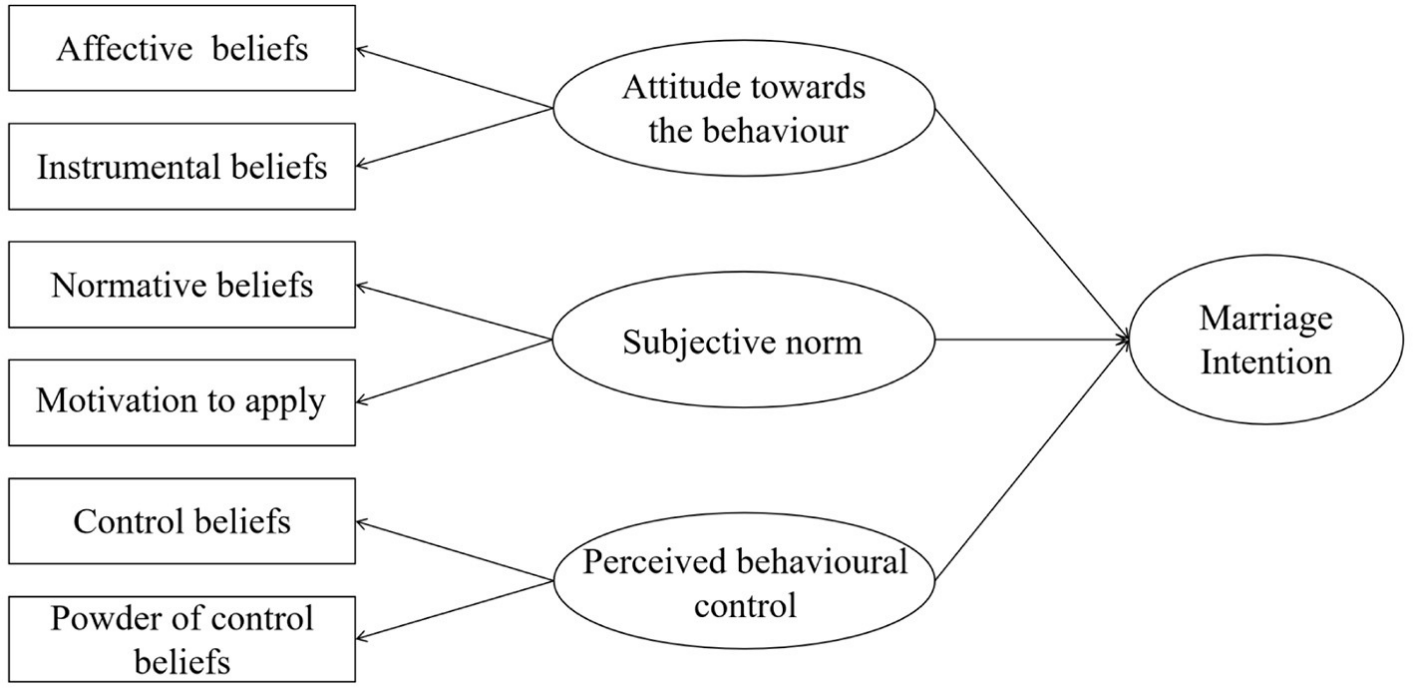
Theoretical model of marriage intention based on the theory of planned behaviour.

Behaviour intention comprises three variables: attitude towards behaviour, subjective norm, and perceived behavioural control (Ajzen, 1991). Among them, attitude towards the behaviour is a psychological experience in which an individual likes or dislikes a specific object and makes a favourable or unfavourable evaluation of the implementation of a specific behaviour, which includes affective and instrumental beliefs (Guo et al., 2013). Subjective norm refers to perceived social pressure when an individual performs a specific behaviour. It reflects the influence of important people or organisations on individual behaviour decision-making, including normative beliefs and motivation to apply. Perceived behavioural control refers to the degree to which an individual feels controllable when performing a specific behaviour, and will be affected by the factors that promote and hinder behaviour performance, that is, control beliefs, and the importance of these factors. Generally speaking, the more favourable the attitude and subjective norm, and the greater the perceived behavioural control, the stronger the individual's intention to perform a specific behaviour, and vice versa. Based on this theory, this study constructed a theoretical model of marriage intention, as shown in Figure 1. Based on the theory of planned behaviour, this study proposes the following hypotheses:

*Hypothesis 1: Attitude towards behaviour has a significant positive impact on the marriage intention of Chinese college students*.

*Hypothesis 2: Subjective norm has a significant positive impact on the marriage intention of Chinese college students*.

*Hypothesis 3: Perceived behavioural control has a significant positive impact on the marriage intention of Chinese college students*.

From an economics perspective, Becker and Gary (1992) proposes ‘specialisation and trading' marriage hypothesis, which believes that the real reason for the existence of marriage is that men and women with different professional advantages and great differences in ability and income can maximise their own and both sides' benefits through marriage. Under this theoretical framework, the improvement of women's economic independence may reduce the marriage benefits brought by spouses, and they tend to make use of their high social and economic status to ‘buy out' marriages that require many opportunity costs (Oppenheimer, 1988, 1994, 1997). Therefore, it is common for women with high socioeconomic status to remain unmarried. Many studies have found that most of the housework and childcare work of heterosexual marriage are still the responsibility of women. Women put in more mental and emotional effort towards family responsibilities than men, and this inequality can easily lead to women's low marriage intention and even marriage refusal. Female college students are well-educated and have many opportunities to gain high socioeconomic status. Considering the economic value and inequality of marriage, we propose the following hypothesis:

*Hypothesis 4: Female college students' marriage intention is lower than that of men*

## Materials and Methods

### Questionnaire Design

Two questionnaires were designed for the research. The first questionnaire was used to elicit the salient beliefs among college students. Before constructing the theory of planned behaviour questionnaire, eliciting the salient beliefs of the investigated population is required. It is assumed that attitude, subjective norm, and perceived behavioural control are based on the corresponding sets of beliefs. Salient beliefs, which means beliefs the most commonly held in the investigated population, play an explanatory function of intentions and behaviours (Ajzen, 1991). In addition, behavioural interventions must try to change a person's salient beliefs. Therefore, an online free-response formatting questionnaire was designed to elicit accessible beliefs among Chinese college students (N = 50; 65% women) from Wenzhou Medical University, Wenzhou University, and Zhejiang Industry and Trade Vocational College by convenient sampling. In detail, the behavioural outcomes were accessed by four questions: “What do you see as the advantages of marriage,” “What do you see as the disadvantages of marriage,” “What positive feelings do you associate with marriage,” “What negative feelings do you associate with marriage.” Normative referents were accessed by four questions: “Please list the individuals or groups who would think you should get married,” “Please list the individuals or groups who would think you should not get married,” “Please list the individuals or groups who are most likely to get married,” “Please list the individuals or groups who are most unlikely to get married.” Control factors were accessed by two questions: “What kind of circumstances that would make it easy for you to get married,” “What kind of circumstances that would make it difficult for you to get married.” A content analysis of the responses to the above questions was conducted and obtained a list of salient beliefs.

Based on the salient beliefs elicited from Chinese college students and literature reviews, 45 questions were designed. After expert revision, questionnaire pilot, validity and reliability test, the final version included 8 questions for basic and demographic characteristics, 27 questions for theory of planned behaviour. 27 questionnaires were subdivided into 7 variables, which are shown in detail in Table 1. Affective beliefs and instrumental beliefs were measured by four items respectively (Shahrabadi et al., 2017). Normative beliefs, motivation to apply, control beliefs, and power of control beliefs were all measured by four items respectively. Marriage intention was measured by three items (Peter and Olson, 1987; Li, 2021). It was designed based on a 7-point Likert scale, where 1 represented strongly disagree, 7 represented strongly agree.

**Table 1:** Cronbach's alpha values and exploratory factor analysis.

**Variable**	**Measurement**	**Factor loading**	**Cronbach's alpha**
Affective beliefs	Marriage will give me a companion for spiritual support	0.774	0.850
	Marriage will give me a sense of belonging	0.794	
	Marriage will bring me happiness	0.778	
	Marriage will make me have quarrels with family members and feel sad	0.667	
Instrumental beliefs	Marriage will give me a partner for material support	0.808	0.880
	Marriage will make me bear children	0.807	
	Marriage will ensure better social relationships with others	0.798	
	Marriage will cause me to lose my personal time and freedom	0.739	
Normative beliefs	My parents think that I should get married	0.847	0.910
	My siblings think that I should get married	0.831	
	My senior relatives think that I should get married	0.787	
	My friends think that I should get married	0.814	
Motivation to apply	How much do you care what your parents think you should do	0.814	0.894
	How much do you care about what your siblings think you should do	0.844	
	How much do you care about what your senior relatives think you should do	0.830	
	How much do you about care what your friends think you should do	0.828	
Control beliefs	I will have good financial status in the future	0.804	0.857
	I will have a stable job in the future	0.804	
	I will have a clear plan about marriage	0.759	
	I will be exposed to positive opinions on marriage by the public	0.703	
Power of control beliefs	If I have a good financial status in the future, it will be easier for me to get married	0.771	0.890
	If I have a stable job in the future, it will be easier for me to get married	0.775	
	If I have a clear plan about marriage, it will be easier for me to get married	0.767	
	If I am exposed to negative opinions on marriage by the public, it will be difficult for me to get married	0.752	
Marriage intentions	It is necessary for me to get married	\	0.835
	I will try to get married	\	
	I plan to get married	\	

### Participants

Chinese college students from Wenzhou Medical University, Wenzhou University, and Zhejiang Industry and Trade Vocational College were selected as research participants by convenience sampling. A total of 850 online questionnaires were distributed: 78 invalid questionnaires were excluded (students who have no intention of marriage were excluded) and 772 valid data were collected (mean age = 20.38 ± 2.38 years; 64.5% women). Regarding education level, the participants included 16.8% junior college students, 56.5% undergraduates, and 26.7% postgraduates.

### Ethics Statement

This study was approved by the ethics committee of Wenzhou Medical University. All participants were informed and provided written consent to participate.

## Data Analysis and Results

### Reliability and Validity Analysis

To investigate whether the measurement items of all variables were highly consistent, IBM SPSS 25 package was used for reliability analysis. The results showed that the alpha values of all variables ranged from 0.835–0.910 (Table 1). This demonstrated that the reliability of the questionnaire was relatively high, and the data were reliable. The KOM value was 0.932 and the chi-square value of Bartlett's statistics was 7436.793, indicating that there was a strong linear relationship between the question items, which was suitable for exploratory factor analysis. The factor loading values were all over 0.5, indicating no cross-factor phenomenon.

### Descriptive Statistical Analysis

Demographic characteristics were collected, including age, sex, and family financial condition. The questions included the appropriate age to marry and how many times participants have been ‘in love'. The most common response regarding the suitable age to get married was 25–27 years (37.09%), followed by 28–30 years (32.67%). Only 6.62% of the students thought that the suitable age was over 30 years. Further, 41.28% of the students had never been in love. There was no significant relationship between financial condition and being in love with the intention to get married.

Table 2 presents the descriptive statistical analysis of measurement items. The mean value of three questions on marriage intention of Chinese college students was 4.38, indicating that they were comparatively inclined to get married when it was time. The highest mean score for intentions was ‘I plan to get married', whereas the lowest mean score was ‘It is necessary for me to get married. In addition, there was a sex difference in marriage intention. The mean value of women's marriage intention was 4.2, whereas that of men was 4.7, indicating that female college students' marriage intention was lower than that of male college students' intention.

**Table 2:** Descriptive statistics.

**Variable**	**Measurement**	**Min**	**Max**	**Mean**	**SD**
Affective beliefs	Marriage will give me a companion for spiritual support	1	7	4.03	1.64
	Marriage will give me a sense of belonging	1	7	3.84	1.72
	Marriage will bring me happiness	1	7	3.89	1.60
	Marriage will make me have quarrels with family members and feel sad	1	7	3.47	1.54
Instrumental beliefs	Marriage will give me a partner for material support	1	7	3.85	1.57
	Marriage will make me bear children	1	7	3.43	1.79
	Marriage will ensure better social relationships with others	1	7	3.88	1.61
	Marriage will cause me to lose my personal time and freedom	1	7	3.53	1.56
Normative beliefs	My parents think that I should get married	1	7	4.69	1.43
	My siblings think that I should get married	1	7	4.28	1.79
	My senior relatives think that I should get married	1	7	4.74	1.76
	My friends think that I should get married	1	7	3.96	1.57
Motivation to apply	How much do you care about what your parents think you should do	1	7	5.03	1.36
	How much do you care about what your siblings think you should do	1	7	4.42	1.46
	How much do you care about what your senior relatives think you should do	1	7	4.04	1.37
	How much do you care about what your friends think you should do	1	7	4.51	1.51
Control beliefs	I can have good financial status in the future	1	7	3.89	1.31
	I can have a stable job in the future	1	7	4.58	1.23
	I will have a clear plan about marriage	1	7	4.08	1.46
	I will be exposed to positive opinions on marriage by the mass media	1	7	3.11	1.41
Power of control beliefs	If I have a good financial status in the future, it will be easier for me to get married	1	7	4.39	1.38
	If I have a stable job in the future, it will be easier for me to get married	1	7	4.44	1.36
	If I have a clear plan about marriage, it will be easier for me to get married	1	7	3.74	1.69
	If I am exposed to negative opinions on marriage by the mass media, it will be difficult for me to get married	1	7	3.84	1.63
Marriage intentions	It is necessary for me to get married	1	7	3.68	1.74
	I will try to get married	1	7	4.72	1.75
	I plan to get married	1	7	4.75	1.90

*Min, minimum; Max, maximum; SD, Standard deviation*.

The mean values of questions on attitudes towards behaviours (affective beliefs and instrumental beliefs), subjective norms (normative beliefs and motivation to apply), and perceived behavioural control (control beliefs and power of control beliefs) were 3.74, 4.46, and 4.00, respectively. The highest mean score for attitude was ‘marriage will make me have a companion for spiritual support', whereas the lowest mean score was ‘marriage will make me bear children'. Students' attitudes towards affective beliefs were slightly more favourable than instrumental beliefs. In terms of subjective norms, important seniors, such as parents and relatives, were more inclined to suggest that students get married than peers, siblings, and friends. For marriage decisions, they were more likely to comply with their parents' suggestions, followed by friends, siblings, and senior relatives. Concerning perceived behavioural control, they were most confident in having stable work in the future, while most diffident in that the mass media would disseminate a positive opinion on marriage. They believed that good financial conditions, stable work, a clear plan, and positive opinions on marriage expressed by the mass media will make it easier to get married, whereas good financial conditions and stable work were more important.

### Structural Equation Model Analysis

AMOS 20.0 statistical software was adopted to test the conceptual model of Chinese college students' marriage intention, verifying the path of attitude towards behaviours (affective beliefs and instrumental beliefs), subjective norms (normative beliefs and motivation to apply), and perceived behavioural control (control beliefs and power of control beliefs) to intention. The fitting index results of the model were χ^2^/df = 1.53, adjusted goodness of fit index (AGFI) = 0.903, goodness of fit index (GFI) = 0.922, Tucker–Lewis index (TLI) = 0.974, Normed Fit Index (NFI) = 0.938, comparative fit index (CFI) = 0.978, root mean square error of approximation (RMSEA) = 0.035, indicating that there was no incorrect setting in the model. The results of the structural equation model analysis in Table 3 illustrated that there was a significant positive correlation between affective beliefs, instrumental beliefs, normative beliefs, motivation to apply, control beliefs, power of control beliefs, and marriage intention.

**Table 3:** Structural equation model analysis.

**Hypothesis path**	**Estimate**	**S.E**.	**C.R**.	**p**	**Fit index**
Affective components → Intention	0.147	0.055	2.302	0.021	χ^2^/df = 1.53
Instrumental components → Intention	0.153	0.050	2.724	0.006	AGFI = 0.903
Motivation to apply → Intention	0.177	0.041	3.532	<0.001	GFI = 0.922
Normative beliefs → Intention	0.110	0.037	2.047	0.041	TLI = 0.974
Control beliefs → Intention	0.129	0.056	2.199	0.028	NFI = 0.938
Power of control beliefs → Intention	0.284	0.055	4.409	<0.001	CFI = 0.978 RMSEA = 0.035

*AGFI, adjusted goodness of fit index; GFI, goodness of fit index; TLI, Tucker–Lewis index; NFI, Normed Fit Index; CFI, comparative fit index, RMSEA, root mean square error of approximation*.

## Discussion

This study applied the theory of planned behaviour in the research of marriage intention, enriching the explanatory and predictive power of the theory of planned behaviour, and explored the motivations of Chinese college students to enter marriage in the future. Structural equation model analyses showed that behavioural attitude, subjective norm, and perceived behavioural control all had significant positive impacts on marriage intention, which is consistent with previous findings (Ajzen, 1991; Canova et al., 2020; Liu et al., 2022); thus, hypotheses 1–3 were valid.

In general, the marital intentions of college students were not low. Although they devalued the necessity of marriage, they expressed their willingness to try and plan to get married. Furthermore, the marriage intention of female college students was significantly lower than that of male college students, verifying Hypothesis 4. This study provides empirical evidence for the ‘specialisation and trading' marriage hypothesis (Becker and Gary, 1992). From the perspective of ‘individualism', Beck and Beck-Gernsheim (2002) agreed that education played an important role in improving women's social status and is an important driving force for them to change from ‘living for others' to ‘living for themselves'. Modern women have a different understanding of their gender identity. They accept new beliefs and norms, have a strong sense of equality, and do not agree with the old norm of ‘men are superior to women' (Xu and Huang, 2018). Although men and women have the same right to work, they are still affected by the gender identity assumption that ‘husbands should earn more than wives, men are responsible for making money, and women are responsible for taking care of the family' (Bittman et al., 2003; Pierce et al., 2013). Concerning the family division of labour, women are still required to devote more for that than men. This kind of inequality results in an increasing number of women choosing late or not to get married (Xu and Huang, 2018). To improve the marriage intention of female college students, the inherent gender identity assumptions need to be adjusted.

Regarding attitude, when eliciting college students' salient beliefs about attitudes, affective beliefs and instrumental beliefs were both obtained. Concerning affective beliefs, ‘Marriage will give me a companion for spiritual support' was supported by the largest group of students. This finding was in line with Shahrabadi et al. (2017) research on Iranian university students' marriage intentions. Regarding instrumental beliefs, the first psychological motivation for college students to choose marriage was that marriage can be conducive for having a better social relationship with others, followed by material support. However, the reproduction function was the least valued. This indicates that even though college students are willing to get married, they may not be willing to bear children in the future. Currently, Chinese families are still traditionally child-centred, and children are still the most important in the family. Confucian culture has always advocated that ‘He who excels in study can follow an official career', emphasising the realisation of inter-generational mobility through education and working hard. Therefore, Chinese families, whether rich or poor, have high expectations for their children's education and make substantial investments in it. Recently, the increasing cost of education has reduced the intention of young people to have children. As mentioned before, childbearing and marriage are closely linked (Yu and Xie, 2019), if students' attitudes towards childbearing are more favourable, their marriage intention will be improved. Students' attitudes towards marriage were also influenced by disadvantages of marriage, as they mentioned that quarrels between family members would make them feel sad, and marriage would result in personal time loss. It is a common phenomenon in China for newly married couples to live together with their in-laws and raise children together with them; hence, both male and female students expressed their worries about mother-in-law and daughter-in-law relations in the open-formatted questionnaire. Some female students even had concerns about infidelity, domestic violence, divorce, and widowed parenting, which shed light on the fact that women are more cautious about marriage. In conclusion, students' attitudes towards marriage should be inverted to be more positive to increase the marriage rate.

Subjective norms are influential factors for marriage intention. Parents' opinions were regarded as the most influential factor. China's marriage norms are deeply affected by Confucianism (Jones et al., 2011). In China, filial piety is the most important of all virtues, and marriage is the command of parents, the words of a matchmaker. Therefore, parents' attitudes and suggestions are vital for college students' marriage intentions and behaviours. When they were asked to list the individuals or groups who would influence them to get married, the most salient individuals or groups were family members, including parents, siblings, and elderly relatives. This highlights the fact that individuals in China are strongly bound to families, and family members have a great impact on personal decisions. In addition, college students also think highly of their friends' opinions. They are willing to accept their friends' suggestions. According to the descriptive statistical analysis, we could see that older generations, such as parents and senior relatives, prefer to experience married life more than the younger generation. It also reveals that elderly people value marriage more highly than young people. The traditional view of marriage and love described by the Chinese saying ‘A man should get married on coming of age, and so should a girl' emphasises that marriage is an indispensable stage of life or even an obligation. However, with the arrival of the second demographic transition, the values and life orientations of the younger generation have changed. Affecting the increasing social tolerance of premarital sex, gender egalitarianism, individualism, materialisation, and attraction of marriage to young people is decreasing. Marriage has become a purposeful personal choice rather than a necessity of life (Lesthaeghe, 1995, 2010; Surkyn and Lesthaeghe, 2004). With the rapid development and progress of society, even if the traditional view of marriage and family still affects the marital choices of the younger generation, this influence will show a weaker and weaker trend.

Concerning perceived behavioural control, participants believed that a good financial condition, stable work, a clear plan, and positive opinions on marriage expressed by the media would make it easier for them to get married, among which a good financial condition and stable work were considered more important. In the Chinese context, unaffordable housing prices, expensive wedding betrothal gifts and banquets, and high children's education costs are all adverse factors impeding young people from married life (Yu and Xie, 2013). Therefore, students generally believe that good economic conditions and stable work are critical for marriage. In addition, college students were dissatisfied with massive negative reports on marriage by the media. The participants of this study were ‘Z-generation youth', which refers to the generation born between 1995 and 2009, also known as the ‘Internet generation'. The combination of the Internet and media magnifies the communication strength and influence of the media. With the development and popularisation of the Internet, the ‘Z-generation' youth get much of their information about marriage online. To attract attention, the mass media prefers to report negative news about marriage, such as the infidelity of celebrities, domestic violence, divorce, and so on. This kind of scattered and unsystematic marriage information contributes to form irrational opinions about marriage, aggravating the unfavourable attitude towards marriage, and even resulting in the phenomenon of ‘fear of marriage' (Kong and Zang, 2021).

This study provides implications for policies and plans to promote marriage rates. According to the theory of planned behaviour, the more favourable the attitude and subjective norm, and the stronger the perceived behavioural control, the greater the individual's intention to perform a specific behaviour, and vice versa. Therefore, according to the above analysis, to facilitate college students' favourable attitude towards marriage, the most salient belief that needs to be adjusted is students' negative attitudes towards fertility. Policies conducive to fertility should be formulated. At present, China has a compliment three-child policy, extended maternity leave, and ‘double reduction' policy to reduce the cost of education. Concerning subjective norms, the family has a great impact on individual decisions. The traditional family view should be better transmitted to college students through their elders, and a harmonious family atmosphere should be created to stimulate college students' yearning for marriage experience. In addition, good economic prospects, more employment opportunities, and positive publicity of marriage by the mass media will also improve college students' marriage intentions. Lastly, given that women's intention to marry is significantly lower than that of men, it is necessary for universities to offer courses on marriage and love to change college students' stereotypical gender identity, meanwhile the curriculum design of courses need to select teaching materials, pedagogical approaches and learning objectives according to contemporary college students' characteristics (Wu and Chen, 2021).

## Conclusion

The contribution of this study is mainly reflected in the following two aspects: at the theoretical and practical level. The marriage intention conceptual model is constructed in this study, which extends and expands the application of the theory of planned behaviour. At a practical level, this study analyses the key predictors affecting college students' marriage intentions and provides practical implications for pro-marriage policymaking.

This study had the following limitations. First, there is evidence that behavioural intention is significantly correlated with actual behaviours (Fishbein and Ajzen, 2010), while marriage intention is affected by actual behavioural control, and the ability to predict marriage behaviour is restrained. Longitudinal research can be conducted in future research to verify the correlation between marriage intention and marriage behaviour. Second, the predictors of marriage intention are selected by eliciting the salient beliefs of college students, and previous research may not be comprehensive. Future research can be conducted to identify other factors affecting marriage intention, including subjective well-being, house pricing, and cohabitation rates. Third, this study was conducted in the Chinese context, and the results cannot be applied to other countries' contexts. Future research should be conducted in different country contexts to investigate the factors affecting marriage intention in different cultures.

## Data Availability Statement

The raw data supporting the conclusions of this article will be made available by the authors, without undue reservation.

## Ethics Statement

This study was approved by the Ethics Committee of Wenzhou Medical University. All participants were informed and provided written consent to participate.

## Author Contributions

JX designed the questionnaires and completed data collection and article writing. XH provided important instructions for the study and suggested revisions. Both authors contributed to the manuscript and approved the submitted version.

## Conflict of Interest

The authors declare that the research was conducted in the absence of any commercial or financial relationships that could be construed as a potential conflict of interest.

## Publisher's Note

All claims expressed in this article are solely those of the authors and do not necessarily represent those of their affiliated organizations, or those of the publisher, the editors and the reviewers. Any product that may be evaluated in this article, or claim that may be made by its manufacturer, is not guaranteed or endorsed by the publisher.
